# Survival Outcomes and Effect of Early vs. Deferred cART Among HIV-Infected Patients Diagnosed at the Time of an AIDS-Defining Event: A Cohort Analysis

**DOI:** 10.1371/journal.pone.0026009

**Published:** 2011-10-17

**Authors:** Jose M. Miro, Christian Manzardo, Cristina Mussini, Margaret Johnson, Antonella d'Arminio Monforte, Andrea Antinori, M. John Gill, Laura Sighinolfi, Caterina Uberti-Foppa, Vanni Borghi, Caroline Sabin, and Late Presenters Investigators

**Affiliations:** 1 Hospital Cliníc-IDIBAPS, University of Barcelona, Barcelona, Spain; 2 Clinic of Infectious and Tropical Diseases, University of Modena and Reggio Emilia, Modena, and Azienda Policlinico, Modena, Italy; 3 Ian Charleson Centre, Royal Free Hospital, London, United Kingdom; 4 Clinic of Infectious Diseases, San Paolo University Hospital, Milan, Italy; 5 National Institute for Infectious Diseases ‘L. Spallanzani’, IRCCS, Rome, Italy; 6 Southern Alberta Clinic, Calgary, Canada; 7 Department of Infectious Diseases, S. Anna Hospital, Ferrara, Italy; 8 Department of Infectious Diseases, Università Vita e Salute, Milan, Italy; 9 UCL Medical Centre, London, United Kingdom; McGill University AIDS Centre, Canada

## Abstract

**Objectives:**

We analyzed clinical progression among persons diagnosed with HIV at the time of an AIDS-defining event, and assessed the impact on outcome of timing of combined antiretroviral treatment (cART).

**Methods:**

Retrospective, European and Canadian multicohort study.. Patients were diagnosed with HIV from 1997–2004 and had clinical AIDS from 30 days before to 14 days after diagnosis. Clinical progression (new AIDS event, death) was described using Kaplan-Meier analysis stratifying by type of AIDS event. Factors associated with progression were identified with multivariable Cox regression. Progression rates were compared between those starting early (<30 days after AIDS event) or deferred (30–270 days after AIDS event) cART.

**Results:**

The median (interquartile range) CD4 count and viral load (VL) at diagnosis of the 584 patients were 42 (16, 119) cells/µL and 5.2 (4.5, 5.7) log_10_ copies/mL. Clinical progression was observed in 165 (28.3%) patients. Older age, a higher VL at diagnosis, and a diagnosis of non-Hodgkin lymphoma (NHL) (vs. other AIDS events) were independently associated with disease progression. Of 366 patients with an opportunistic infection, 178 (48.6%) received early cART. There was no significant difference in clinical progression between those initiating cART early and those deferring treatment (adjusted hazard ratio 1.32 [95% confidence interval 0.87, 2.00], p = 0.20).

**Conclusions:**

Older patients and patients with high VL or NHL at diagnosis had a worse outcome. Our data suggest that earlier initiation of cART may be beneficial among HIV-infected patients diagnosed with clinical AIDS in our setting.

## Introduction

Despite the fact that combined antiretroviral treatment (cART) has made it possible to control HIV infection and extend survival if started with a CD4+ cell count above 200 cells/mm^3^ [1]–[2], a considerable proportion of patients still present with an AIDS-defining condition at diagnosis [3]–[8]. Following treatment guidelines, these patients should start cART. For AIDS-defining conditions that do not have a specific treatment, such as progressive multifocal leukoencephalopathy (PML) or cryptosporidiosis, cART should be started as soon as possible. However, for AIDS-defining conditions that do have a specific treatment, the possibility of overlapping toxicities and pharmacokinetic/pharmacodynamic interactions between cART and other drugs, and the possibility of developing inflammatory immune reconstitution syndrome (IRIS) [9]–[10], make it difficult to decide whether to start cART immediately or defer it until the patient's condition stabilizes [11]. The timing of cART in this case has not been well established, and current guidelines are based on observational studies and expert opinion.

The ACTG A5164 trial [12] demonstrated that early cART resulted in less rapid AIDS progression and death than deferred cART. However, as 75% of the study sample had *Pneumocystis jiroveci* pneumonia (PCP) and bacterial infections, these results may not be valid for patients with other OIs or AIDS-defining conditions that were underrepresented in this study or for patients with tuberculosis, who were excluded. In addition, as the health-care systems function differently in resource-limited settings, the results of trials conducted in these settings may not be applicable to other settings, and contradictory results may be obtained. For example, in patients with cryptococcal meningitis, early cART reduced mortality in the ACTG trial [12] but was deleterious in a trial performed in Zimbabwe [13]. Regarding tuberculosis (TB), randomized clinical trials (RCTs) conducted in resource-limited settings [14]–[17] suggest that early cART (initiated during the first 15 days) may lead to benefit among HIV-infected, cART-naïve patients with TB, at least for those with the greatest level of immunosuppression (baseline CD4+ T cells <50 cells/mm^3^). As RCTs of the timing of cART in patients with OIs are likely to be infeasible in developed countries because of the low incidence of OIs, observational data from different clinical settings in Western countries may provide insight as to whether it is possible to extrapolate the information obtained from RCTs conducted in resource-limited countries and may help physicians to improve the clinical management of advanced patients.

The aims of this study were to analyze clinical progression and survival among persons diagnosed with HIV infection at the time of an AIDS-defining event and to assess the outcome of timing of cART (early *versus* deferred) in these individuals in eight cohorts from Europe and Canada.

## Methods

This is a retrospective, multinational, multicohort study. We pooled data on patients newly diagnosed with HIV presenting with a clinical AIDS-defining event from 1997 to 2004 at the following centers: Clinic of Infectious Diseases, Modena, Italy; Department of Infectious Diseases, Ferrara, Italy; Clinic of Infectious Diseases, Milan, Italy; Infectious Diseases Department, San Raffaele Hospital, Milan (HSR), Italy; INMI Spallanzani, Rome, Italy; Infectious Diseases Service, Hospital Clinic – IDIBAPS, University of Barcelona, Barcelona, Spain; the Ian Charleson Centre, Royal Free Hospital, London, UK; Southern Alberta Clinic, Calgary, Canada. This study was approved by Institutional Review Boards (IRBs) of all the above listed hospitals and IRBs specifically approved the use of data without the informed consent of the subjects, since data were analyzed retrospectively and anonymously.

Eligible patients from each cohort were those who had an AIDS diagnosis (defined using the Centers for Disease Control and Prevention (CDC) criteria) from 30 days before until 14 days after their first positive HIV test. All AIDS-defining events occurring during this period were considered to be events that occurred at the time of diagnosis; any AIDS-defining event that occurred more than 14 days after HIV diagnosis was considered to be a subsequent event. Datasets were requested in a common format and included demographic data (date of birth, sex, and risk group), clinical events (dates and type of all AIDS events, date of death, and date of HIV diagnosis), laboratory measurements at diagnosis and during follow-up (CD4 cell counts and percentages, CD8 cell counts and percentages, HIV RNA levels and haemoglobin), and information on antiretroviral use (dates of starting and stopping all antiretroviral drugs).

Early cART was defined as treatment started within the first 30 days after the diagnosis of the AIDS-defining condition; deferred cART was defined as treatment started between days 31 and 270 days after diagnosis of the AIDS-defining condition. The choice of 30 days as cut off was made *a priori* and based on our clinical experience as well as on results of several randomized clinical trials (e.g. in the ACTG A5164 study [12] patients in the deferred arm started cART from 28–244 days after diagnosis). Patients who died or were lost to follow-up between day 0 and day 30 after diagnosis of their AIDS-defining condition were excluded from the analysis, regardless of when cART was initiated; patients dying between days 31 and 270 without starting cART were assigned to the deferred group. For analysis purposes, AIDS-defining events were categorized as tuberculosis (TB), PCP, Kaposi's sarcoma (KS), non-Hodgkins lymphoma (NHL), and other OIs. Clinical progression was defined as a new AIDS-defining event or death (combined endpoint).

### Statistical analysis

Patient follow-up was counted from the date of HIV diagnosis until the date of the first new AIDS event (more than 14 days after diagnosis) or death, whichever occurred first. Follow-up on patients who remained alive and without a new AIDS event at the end of the study period was right-censored on the date of the patient's last CD4 count or HIV viral load, whichever was latest. Kaplan-Meier plots were used to describe progression rates among all patients after stratifying by the type of AIDS event that was present (see previous section). Factors associated with clinical progression were identified using univariable and multivariable Cox proportional hazards regression models. Factors were considered to be statistically significant if p<0.05.

Clinical progression rates were compared in the early and deferred cART groups using a similar approach. These analyses were restricted to the 366 patients presenting with an opportunistic infection who could be classified into one of these two groups. Follow-up for these analyses again started at the time of HIV diagnosis, with adjustment for sex, risk group, age, CD4 count and viral load at diagnosis. Analyses were repeated considering progression to death alone, and among the subgroups of patients presenting with PCP and TB.

## Results

### Analyses of clinical progression according to type of AIDS event

**Figure 1:**
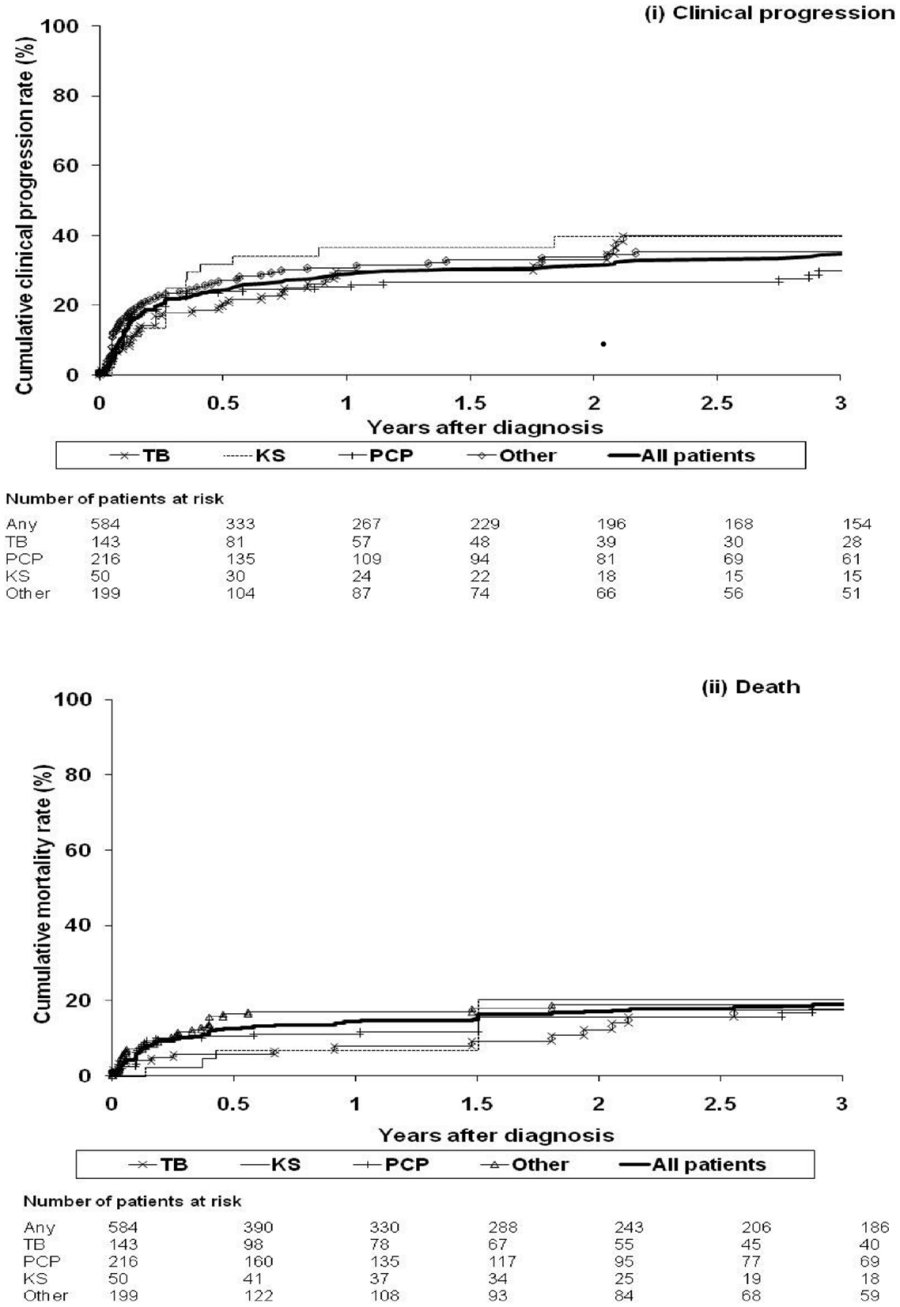
Kaplan-Meier plot of the cumulative proportion of patients experiencing (i) clinical progression and (ii) death, according to the AIDS diagnosis present at HIV diagnosis*. * As patients may have had more than one AIDS-defining condition at the time of diagnosis, they may fall into more than one of the groups in the figure.

A total of 584 patients were included in these analyses. **Table 1** shows selected characteristics of the study patients overall and stratified by AIDS-defining event. Of the 584 patients, 96 (16.4%) were known to have died, 110 (18.8%) experienced a new AIDS event, and 165 (28.3%) experienced the combined clinical progression endpoint (new AIDS event or death). **Figure 1** shows the proportion of patients experiencing clinical progression and dying both overall and stratified by initial AIDS-defining event. Overall, progression rates at 3, 6, 9, and 12 months were 20.3%, 24.4%, 27.3%, and 28.8%; mortality rates at the same timepoints were 9.5%, 12.5%, 13.6% and 14.4%, respectively. Of note, almost all patients who experienced clinical progression or death did so during the first year of follow-up, with patients with a diagnosis of NHL at baseline having a particularly poor prognosis.

**Table 1:** Patient characteristics, overall and stratified by type of AIDS-defining event*; entries are n (%) unless otherwise stated.

		All patients	TB	PCP	KS	NHL	Other OIs**
N (% of total)		584	143	216	50	15	199
Male sex		446 (76.5)	101 (71.1)	168 (77.8)	46 (92.0)	12 (80.0)	150 (75.4)
Risk group	IDU	77 (13.2)	32 (22.4)	22 (10.2)	0 (-)	0 (-)	20 (10.1)
	Homosexual	118 (20.2)	12 (8.4)	63 (29.2)	17 (34.0)	2 (13.3)	42 (21.1)
	Heterosexual	190 (32.5)	61 (42.7)	75 (34.7)	13 (26.0)	5 (33.3)	57 (28.6)
	Other	199 (34.1)	38 (26.6)	56 (25.9)	20 (40.0)	8 (53.3)	80 (40.2)
Days from AIDS to HIV diagnosis	Median (IQR)	0 (−2, 2)	0 (−1, 6)	0 (−2, 1)	0 (−3, 0)	0 (−5, 0)	0 (−1, 0)
Age at diagnosis (years)	Median (IQR)	39 (33, 48)	37 (31, 43)	39 (34, 47)	48 (34, 54)	40 (39, 49)	40 (34, 51)
CD4 count at diagnosis (cells/mm^3^)	Median (IQR)	40 (14, 109)	81 (25, 177)	27 (11, 68)	84 (15, 231)	109 (66, 195)	27 (10, 78)
HIV RNA at diagnosis (log_10_ cp/ml)	Median (IQR)	5.3 (4.6, 5.7)	5.4 (4.9, 5.9)	5.2 (4.5, 5.6)	5.4 (4.5, 5.7)	5.0 (4.4, 5.4)	5.2 (4.6, 5.7)
Country of origin	Italy	222 (38.0)	36 (25.2)	80 (37.0)	27 (54.0)	8 (53.3)	77 (38.7)
	Spain	175 (30.0)	58 (40.6)	53 (24.5)	7 (14.0)	0 (-)	64 (32.2)
	UK	132 (22.6)	45 (31.5)	49 (22.7)	13 (26.0)	4 (26.7)	38 (19.1)
	Canada	55 (9.4)	4 (2.8)	34 (15.7)	3 (6.0)	3 (20.0)	20 (10.1)
Started cART		477 (81.7)	115 (80.4)	179 (82.9)	47 (94.0)	13 (86.7)	160 (80.4)
Days to start of cART	Median (IQR)	31 (13, 53)	42 (18, 79)	30 (15, 43)	18 (1, 37)	44 (21, 61)	27 (10, 49)
Timing of cART***	Immediate	211 (50.5)	39 (41.9)	85 (50.0)	30 (69.8)	3 (30.0)	72 (51.4)
	Deferred	207 (49.5)	54 (58.1)	85 (50.0)	13 (30.2)	7 (70.0)	68 (48.6)
Type of cART	NNRTI-based	137 (28.7)	44 (38.3)	47 (26.3)	12 (25.5)	4 (30.8)	46 (28.8)
	PI-based	297 (62.3)	54 (47.0)	124 (69.3)	32 (68.1)	9 (69.2)	95 (59.4)
	Other	43 (9.0)	17 (14.8)	8 (4.5)	3 (6.4)	0 (-)	19 (11.9)
Total follow-up (years)	Median (IQR)	1.5 (0.2, 3.4)	1.4 (0.2, 3.5)	1.6 (0.4, 3.5)	2.0 (0.9, 3.9)	0.4 (0.0, 0.7)	1.3 (0.1, 3.3)
New AIDS event within first year§		110 (18.8)	31 (21.7)	36 (16.7)	18 (36.0)	3 (20.0)	39 (19.6)
Death within first year§		70 (12.0)	9 (6.3)	22 (10.2)	3 (6.0)	7 (46.7)	28 (14.1)

*As patients may have experienced more than one of the specific AIDS-defining events, each event is treated as a separate binary covariate.

**Included the following events: oesophageal candidiasis (n = 75); toxoplasmosis (n = 50); cytomegalovirus (n = 36); cryptococcal meningitis (n = 19); cryptosporidiosis (n = 8); progressive multifocal leukoencephalopathy (n = 18), herpes simplex infections (n = 10).

***Based on patients with sufficient follow-up to be categorized into one of the two groups (see Methods).

§ A small number of patients developed AIDS-defining conditions or died that did not fall into one of the five specific categories (e.g. wasting syndrome) – these patients are included in the total column but will not be in the subcolumns.

Unadjusted analyses (**Table 2**, left-hand column) confirmed the poorer prognosis among those with NHL, with these individuals being more than twice as likely to experience clinical progression (relative hazard [RH] 2.38 [95% confidence interval 1.08, 5.23], p = 0.03) than individuals with other AIDS diagnoses. Other factors associated with a poorer prognosis were older age (RH per 5 years older: 1.14 [1.06, 1.22], p = 0.0003) and a higher viral load at diagnosis (RH per log_2_ increment: 1.32 [1.10, 1.58], p = 0.002). The poorer prognosis among those with NHL remained after adjustment for age and viral load (**Table 2**, right-hand column).

**Table 2:** Factors associated with clinical progression (a new AIDS event or death) (N = 584).

	Univariable models			Multivariable model		
	RH	95% CI	p-value	RH	95% CI	p-value
AIDS diagnosis*						
TB vs. no TB	1.00	(0.67, 1.49)	0.99	1.17	(0.73, 1.86)	0.51
PCP vs. no PCP	0.83	(0.58, 1.18)	0.29	0.94	(0.63, 1.41)	0.77
KS vs. no KS	1.12	(0.66, 1.88)	0.68	1.32	(0.77, 2.26)	0.32
NHL vs. no NHL	2.38	(1.08, 5.23)	0.03	2.84	(1.11, 7.23)	0.03
Female	1.06	(0.74, 1.53)	0.75	-	-	-
Risk group						
Heterosexual	1	-	-	-	-	-
Homosexual	0.63	(0.39, 1.01)	0.06	-	-	-
IDU	1.15	(0.71, 1.86)	0.56	-	-	-
Other	1.20	(0.83, 1.73)	0.34	-	-	-
Age at diagnosis (/5 years older)	1.14	(1.06, 1.22)	0.0003	1.12	(1.03, 1.21)	0.007
CD4 count at diagnosis (/50 cells/mm^3^ higher)	0.96	(0.90, 1.03)	0.24	-	-	-
Viral load at diagnosis (/log_10_ higher)	1.32	(1.10, 1.58)	0.002	1.34	(1.12, 1.60)	0.001

*As patients may have experienced more than one of the specific AIDS-defining events, each event is treated as a separate binary covariate; therefore, patients with the event of interest are compared to those without that event (but who will have at least one of the other AIDS events).

Results of analyses of mortality reached similar conclusions. In unadjusted analyses, patients with a diagnosis of NHL were over four times as likely to die than patients with other AIDS events (RH: 4.23 [1.86, 9.57], p = 0.0006); this association remained (adjusted RH: 9.15 [3.31, 25.31], p = 0.0001) after adjusting for the patient's age and viral load at the time of diagnosis.

### Analyses of clinical progression according to timing of treatment

Of the 584 patients, 159 were not included in these analyses because they did not have sufficient follow-up or data (25 were lost to follow-up between day 0 and 30, 104 had insufficient follow-up to be classified and 30 could not be categorized into one of treatment arms for other reasons). An additional seven patients died within the first 30 days, leaving 418 patients who met the criteria previously defined. Overall, those who were excluded were more likely to be from the injecting drug users group (21% of those excluded vs. 10% of those included) or ‘Other’ (40% vs. 32%) risk groups and were correspondingly less likely to be from the homosexual (10% vs. 24%) risk group (p = 0.0001). Excluded patients were more likely to come from Spain (50% vs. 22%) and less likely to come from the other participating countries (p = 0.0001). Finally, largely as a result of the differences in risk groups, those excluded were more likely to be female (29% vs. 21%, p = 0.07). Baseline age, CD4 T- cells count and plasma HIV RNA levels did not differ significantly between excluded and included patients.

Of the 418 patients, 366 had presented with an opportunistic infection and were included in the analysis: 178 (48.6%) received early treatment and 185 (50.5%) received deferred treatment (**Table 3**). Only three patients (0.8%) never started cART. A total of 75 (20.5%) patients developed a new AIDS event and 18 (4.9%) died, with 90 (24.6%) experiencing clinical progression.

**Table 3:** Patient characteristics, overall and stratified by timing of cART (early *versus* deferred); entries are n (%) unless otherwise stated.

		All patients	Timing of cART		
			Early	Deferred	p-value
N (% of total)		366 (100.0)	178 (48.6)	188 (51.4)	
Male sex		282 (77.3)	132 (74.6)	150 (79.8)	0.29
Risk group	IDU	43 (11.8)	18 (10.1)	25 (13.3)	
	Homosexual	83 (22.7)	37 (20.8)	46 (24.5)	
	Heterosexual	125 (34.2)	65 (36.5)	60 (31.9)	
	Other	115 (31.4)	58 (32.6)	57 (30.3)	0.56
Age (years)	Median (IQR)	39 (33, 46)	39 (33, 48)	38 (33, 43)	0.14
CD4 count at diagnosis (cells/mm^3^)	Median (IQR)	34 (13, 98)	32 (12, 103)	34 (16, 92)	0.73
HIV RNA at diagnosis (log_10_ copies/ml)	Median (IQR)	5.2 (4.6, 5.7)	5.2 (4.4, 5.7)	5.3 (4.8, 5.7)	0.18
Country of origin	Italy	138 (37.7)	78 (43.8)	60 (31.9)	
	Spain	89 (24.3)	39 (21.9)	50 (26.6)	
	UK	97 (26.5)	51 (28.7)	46 (24.5)	
	Canada	42 (11.5)	10 (5.6)	32 (17.0)	0.002
Type of AIDS event	TB	93 (25.4)	39 (21.9)	54 (28.7)	0.17
	PCP	158 (43.2)	75 (42.1)	83 (44.2)	0.78
	Other*	135 (36.9)	68 (38.2)	67 (35.6)	0.69
Days to start of cART		31 (14, 49)	14 (4, 22)	49 (39, 71)	0.0001
Type of cART					
	NNRTI-based	102 (28.1)	44 (24.7)	58 (31.4)	
	PI-based	226 (62.3)	117 (65.7)	109 (58.9)	
	Other	35 (9.6)	17 (9.6)	18 (9.7)	
	No cART	3 (0.8)	-	3 (1.6)	0.35
Total follow-up (years)	Median (IQR)	2.2 (0.9, 4.1)	2.4 (0.8, 4.0)	2.1 (1.0, 4.5)	0.85

*Included the following events: oesophageal candidiasis (n = 59); toxoplasmosis (n = 29); cytomegalovirus (n = 23); cryptococcal meningitis (n = 15); cryptosporidiosis (n = 7); progressive multifocal leukoencephalopathy (n = 5), herpes simplex infections (n = 8).

**Table 3** shows the characteristics of these patients, overall and stratified by early or deferred treatment. The median time for starting cART in the early treatment and deferred treatment groups was 14 (IQR: 4, 22) and 49 (IQR: 39, 71) days, respectively. Overall, the characteristics of patients receiving early and deferred treatment were broadly similar with the exception of some differences by country. There were no significant differences between the treatment regimens in the two groups.

In unadjusted analysis, patients who deferred treatment were 32% more likely to progress to a new AIDS event or die than those initiating treatment early, although this finding was not statistically significant (unadjusted RH 1.32 [0.87, 2.00], p = 0.20); this effect was unchanged (adjusted RH 1.39 [0.90, 2.15], p = 0.14) after controlling for other clinical and demographic characteristics at baseline (sex, risk group, age, CD4 count and viral load at diagnosis). Analyses of progression to death alone reached similar conclusions (unadjusted RH: 1.37 [0.71, 2.65], p = 0.34; adjusted RH: 1.59 [0.77, 3.28], p = 0.21) although as patients who died in the first 30 days after diagnosis (n = 7) were excluded from these analyses, these findings should be interpreted cautiously.

In the subgroup of patients presenting with PCP only (n = 152; 74 (48.7%) early treatment), 31 (20.4%) patients experienced clinical progression. As before, patients who deferred treatment were more likely to experience clinical progression, although differences were not significant, either before or after adjustment for potential confounders (unadjusted RH: 1.68 [0.80, 3.51], p = 0.17; adjusted RH: 1.90 [0.90, 4.03], p = 0.09). In the subgroup of patients presenting with TB only (n = 104; 51 (49.0%) early treatment), 27 (26.0%) patients experienced clinical progression. Again, patients who deferred treatment were more likely to experience clinical progression, with differences reaching statistical significance after adjustment for potential confounders (unadjusted RH: 2.00 [0.90, 4.47], p = 0.09; adjusted RH 2.55 [1.10, 5.94], p = 0.03). On the other hand, 47 patients had more than one AIDS-defining opportunistic infection at the time of diagnosis (two: n = 44; three: n = 3). There were no association between having multiple events and clinical progression (adjusted RH 1.38 [0.78, 2.44], p = 0.27).

Finally, there was no evidence that clinical progression changed over time (RH [95%CI] per later year: 0.97 [0.87, 1.09], p = 0.64) and there was no evidence of any interaction between the treatment effect and the calendar year (p = 0.87).

## Discussion

A considerable proportion of patients continue to present with a CDC category C event at diagnosis of HIV infection either in developed or in developing countries. As the optimal timing for starting cART in this clinical setting has not been well-established, several recent clinical trials [12]–[16] have attempted to clarify this important issue. Our study is based on observational data collected before the publication of these results. Concerns about pharmacokinetic and pharmacodynamic interactions and overlapping toxicities between antiretrovirals and antimicrobial drugs used for treating OIs and the possibility of immune reconstitution syndrome meant that in clinical practice many clinicians preferred to defer cART [11]–[18]. However, recently published data suggests that early cART is crucial for optimal prognosis in patients presenting with an AIDS-defining opportunistic infection [12]–[16]. Data from the ACTG A5164 [12] study suggest that, globally, for fungal infections and/or CD4+ T-cell count lower than 50 cells/µL, an early initiation of cART (median 12 days) significantly reduces risk for disease progression and death compared with a later start of cART (median 45 days), indicating that even a few weeks of earlier cART could be beneficial in antiretroviral-naïve, HIV-infected individuals diagnosed with these opportunistic infections. Authors of this ACTG trial admit a possible selection bias in their study, since patients ‘not ideal’ for a RCT, such as active injecting drug users (IDUs) or patients with very severe infections may have been excluded. A post-hoc analysis of the ACTG A5164 study showed a trend towards a better outcome for patients presenting with PCP starting cART in the early treatment arm, although statistical significance was not reached (p>0.05). Similarly, in our study, patients with PCP who deferred treatment were 90% more likely to progress to a new AIDS event or die than those initiating treatment in the first 30 days after diagnosis, although the difference between the two groups did not reach statistical significance (p = 0.09 in the adjusted analysis).

TB is the second most common OI in some European settings and the most common one worldwide. The RCTs on timing of cART in the context of HIV-TB co-infection have been conducted in research-poor settings [14]–[17], whereas in Western countries only observational data are available [18]. The SAPIT study [14] determined that antiretroviral treatment should be started during the intensive phase or maintenance phase of tuberculosis treatment in patients with pulmonary TB and should not be delayed until after the completion of the latter, at least for patients with CD4+ T-cells counts equal to or lower than 500 cells/µL. A sub-study from this trial [15], presented at the 2011 CROI concluded that among patients with pulmonary TB with a CD4 T-cell count <50 cells/µL, cART initiation within 4 weeks was associated with improved AIDS-free survival, but a higher risk of IRIS; for patients with a CD4+ T-cell count >50 cells/mm^3^ earlier cART initiation was associated with a reduction in the risk of IRIS without compromising AIDS-free survival. As yet unpublished data from the CAMELIA [16] and ACTG 5221 STRIDE [17] studies also confirms that for individuals with severe immunosuppression (CD4+ T cells count <50 cells/mm^3^), early cART (within 2 weeks) significantly reduced the risk of disease progression and death. In our study, the median (IQR) CD4 T cell count for patients co-infected with TB was 81 cells/µL (25–177). In this sub-group, starting cART 30 days after diagnosis carried a significantly higher probability of disease progression or death compared with patients starting cART earlier (adjusted HR 2.55 [1.10, 5.94], p = 0.03). One of the limitations of our study was that we did not know whether the patients presented pulmonary or extra-pulmonary forms of TB. Based on data from the Euro HIV/TB study [19], the prevalence of extra-pulmonary TB in Central/Northern Europe and Southern Europe was 73.8% and 61.9%, respectively. Therefore, most cases from our cohort very probably had extra-pulmonary TB. This is an important issue because of more than 90% of patients included in the three RCTs stated above had pulmonary TB [14]–[17]. For this reason, it would be desirable to have more data regarding the best timing of cART for extra-pulmonary TB in both developed and developing countries [20].

In this way, the clinical scenario is more complex in central nervous system opportunistic infections where early initiation of cART does not always reduce mortality. One randomized trial in Zimbabwe [13] compared the outcome of 54 patients diagnosed with cryptococcal meningitis and treated with fluconazole monotherapy and concluded that early cART was detrimental in this patient group (88% mortality rate in patients treated in the first 72 hours *vs.* 54% in patients who deferred cART for 4 weeks, P< 0.006). In a randomized trial performed in Vietnam [21], early cART (within 2 weeks) did not confer a survival benefit in patients with TB meningitis. Therefore, when the OI (at least cryptococcal and TB) involves the central nervous system early cART could prove an exception in this clinical context; however, these results cannot be extended to resource-rich settings (e.g. early cART reduced mortality in patients with cryptococcal meningitis included in the ACTG trial [12]), and more controlled data are needed in order to confirm them. We did not have information regarding the number of cases of TB meningitis in our European and Canadian cohorts. However, the proportion is probably low. In one study performed in Spain in the pre-cART era, less than 10% of TB cases in HIV-infected patients had central nervous system involvement [22].

Our study shows a trend towards a better outcome for patients presenting with an AIDS-defining condition at diagnosis of HIV infection who started cART in the first 30 days from diagnosis. For OIs other than PCP and TB, a trend towards a better outcome in patients starting cART in the first 30 days was also observed, although specific conclusions can't be reached because of limited sample size and because of the heterogeneity of conditions (including oesophageal candidiasis, toxoplasmosis, cytomegalovirus, cryptococcal meningitis, cryptosporidiosis, progressive multifocal leukoencephalopathy, and herpes simplex infections) that were seen in this group.

Timing of cART has been shown to differ by country (Canada started later, Italy earlier) and by type of AIDS-defining event (in patients presenting with KS, cART was started earlier than in other conditions). cART regimens also were broadly similar in the early and the deferred arms, with some differences depending on type of OI. In our study, the factors independently associated with disease progression and death were a diagnosis of NHL, older age, and a higher viral load at diagnosis. Prognosis was independent of CD4 count stratum, most likely reflecting the universally low CD4 counts in these patients. Of note, where clinical events occurred, they were generally observed in the first 48 weeks of follow-up, suggesting that patients who do not experience clinical progression during this period are likely to have a good subsequent prognosis. Survival analysis showed that patients diagnosed with NHL had the worst prognosis.

### Limitations

Our study has some limitations by the fact that it is a retrospective cohort study and the information at the time of AIDS diagnosis (particularly CD4 count and VL) may sometimes be missing. Despite the large sample size, the number of patients with some AIDS-defining cancers (e.g. KS and NHL) remains low, limiting any conclusions that can be drawn relating to the timing of cART in patients with cancer. As a result, we were unable to perform sub-analyses of OIs other than PCP and tuberculosis. Furthermore, as information was not available on the location of tuberculosis, we were unable to consider tuberculosis meningitis as a specific condition. As this is an observational study, we cannot rule out the possibility of unmeasured confounders (e.g. bias by clinical indication); it is possible that any differences seen between those in the early and deferred cART groups may reflect other unmeasured differences between these patients. Of note, the seven patients who died rapidly were excluded from our analyses; thus, our conclusions are applicable only to those who survive for at least 30 days after HIV diagnosis. Finally, cause of death and reasons for never starting cART remain unknown in some patients.

### Conclusions

In conclusion, the results of our cohort study are consistent with data emerging from randomized clinical trials [12]–[14], [15] and suggest an earlier start for cART among HIV-infected patients diagnosed with an OI-defining event. Patients with PCP at baseline tended to have a better outcome when cART was started earlier. Among individuals with TB, people deferring cART for more than 30 days after diagnosis of their OI had twice the risk of clinical progression or death than those who initiated cART immediately. Since almost all randomized clinical trials for timing of cART were conducted in resource-limited settings, more controlled data are needed to establish the best timing for starting cART in patients presenting with AIDS-defining OI in developed countries, where access to cART and health-care systems function differently.
